# NERChem: adapting NERBio to chemical patents via full-token features and named entity feature with chemical sub-class composition

**DOI:** 10.1093/database/baw135

**Published:** 2016-10-25

**Authors:** Richard Tzong-Han Tsai, Yu-Cheng Hsiao, Po-Ting Lai

**Affiliations:** 1 Department of Computer Science and Information Engineering, National Central University, Taiwan, R.O.C; 2 Department of Computer Science, National Tsing-Hua University, Taiwan, R.O.C

## Abstract

Chemical patents contain detailed information on novel chemical compounds that is valuable to the chemical and pharmaceutical industries. In this paper, we introduce a system, NERChem that can recognize chemical named entity mentions in chemical patents. NERChem is based on the conditional random fields model (CRF). Our approach incorporates (1) class composition, which is used for combining chemical classes whose naming conventions are similar; (2) BioNE features, which are used for distinguishing chemical mentions from other biomedical NE mentions in the patents; and (3) full-token word features, which are used to resolve the tokenization granularity problem. We evaluated our approach on the BioCreative V CHEMDNER-patent corpus, and achieved an F-score of 87.17% in the Chemical Entity Mention in Patents (CEMP) task and a sensitivity of 98.58% in the Chemical Passage Detection (CPD) task, ranking alongside the top systems.

**Database URL:** Our NERChem web-based system is publicly available at iisrserv.csie.n cu.edu.tw/nerchem.

## Introduction

Chemical patents contain descriptions of the inventions of the chemical and pharmaceutical industries, like therapeutic drugs or other functional synthetic compounds. The synthetic compounds and molecules mentioned in the patents are especially valuable for research into organic ingredient synthesis, and there is an increasing demand for assistance in automatically recognizing these substances in the patents.

To meet these needs, the BioCreative Organization Committee organized a CHEMDNER-patent track in 2015 to provide a benchmark for developing and evaluating chemical and protein name recognition systems for chemical patents. The track was separated into three subtasks: the CEMP (Chemical Entity Mention in Patents) subtask, the CPD (Chemical Passage Detection) subtask and the GPRO (gene and protein related object) subtask. All subtasks followed the same annotation platform and chemical classes as the BioCreative IV CHEMDNER track but with some additional rules. Table 1 shows some examples of each class.

**Table 1:** Examples of each class

Type	Example
Abbreviation	“DMSO”, “HAIP”
Family	“spiromethylene”, “halogen”
Formula	“-CH=CH-CH=CH-”, “C9H8O4”
Identifier	“HMQ1611”, “ZSTK474”
Multiple	“vitamin B-6, B12”, “Chemomicin B,C,D”
Systematic	“2-hydroxymethyl-5-(5-fluorocytosin-1-yl)-1,3-oxathiolane”
Trivial	“Azithromycin”, “menthol”

In this study, we developed a conditional random fields (CRF)-based chemical name recognizer, NERChem. Following the guidelines of BioCreative’s CEMP subtask, our system identifies CEMs in sentences but does not classify these mentions into fine-grained CEM types. NERChem attempts to address three potential problems of chemical named entity (CNE) recognition: (1) Chemical named entities can be divided into several classes, each of which has its own naming convention. Many chemical name recognition (CNR) systems (1–3) merged all NE classes into a single class. In our study, we only merge those classes with similar naming conventions. (2) Chemical patent documents may contain biological NEs (BNE) such as genes and cells, and it may be difficult to distinguish CNEs from BNEs. Here, we identify BNEs and use their occurrence as BioNE features on CEMP. (3) Some chemical compound mentions (e.g. 2-hydroxymethyl-5-(5-fluorocytosin-1-yl)-1,3-oxathiolane) contain token delimiters (e.g. ‘−’), which split them into several tokens. Traditional NER systems split such expressions into sequences of tokens. Then, for each token *t*, features corresponding to *t* are extracted and fed to machine-learning models, which predict *t*’s label. However, such tokens are fragments of a full token and lose the actual token’s morphological characteristics. For this reason, we use features extracted from the full tokens instead of features extracted from token fragments.

## Related work

In this section, we review the latest chemical name recognition researches. Most CNR systems (1–4) use machine-learning (ML)-based approaches, and here we examine five aspects of CNR: (1) CNR corpora; (2) tokenization method; (3) ML classifier; (4) tag set; (5) features set.

CNR corpora such as CHEMDNER (5), IUPAC (6) and SCAI (7) have been used by many CNR systems (2–5, 8, 9). The CHEMDNER corpus contains 7000 abstracts in the training set, making it significantly larger than other CNR corpora. It divides the chemical names into several classes including ABBREVIATION, IDENTIFIER, FAMILY, FORMULA, MULTIPLE, SYSTEMATIC, TRIVIAL and NO_CLASS. CHEMDNER-patents (10) and Akhondi et al.’s corpus (11) are CNR corpora of chemical patents. As previously mentioned, CHEMDNER-patents corpus was created using the same annotation platform and chemical classes as the CHEMDNER corpus with some additional rules. It is divided into the training, development, and test sets, each containing 7000 patent abstracts. We use it in this paper because it is the largest available CNR corpus of chemical patents.

To tokenize chemical patent texts, different tokenization methods have been used by different systems. tmChem (1) splits a sentence into tokens by delimiters (e.g. whitespaces, punctuation marks and digits) and by transitions of different character types (e.g. lowercase to uppercase character transition and vice versa). It achieves the best performance on the CHEMDNER corpus. Another state-of-the-art CNR system, CheNER (3), uses whitespaces to divide sentences and punctuation symbols at the end of tokens are removed during token feature extraction. CheNER is a publicly available CNR tool also developed on the CHEMDNER corpus. Batista-Navarro *et al*. (4) used OSCAR4 (12) to tokenize sentences and found that using the OSCAR4 tokenization achieved a higher CNR performance than using the GENIATagger tokenization.

For the ML component, most CNR systems use linear chain CRF tools such as CRF ++ (13) and MALLET (14). For example, tmChem combines two independent NER methods, BANNER-based (15) and tmVar-based (16) CNR systems. The BANNER-based system uses MALLET and the tmVar-based system uses CRF ++.

In the tag set, tmChem uses the SOBIE tag scheme with only one entity label CHEMICAL. Since the chemical corpus has several classes, CheNER tries to recognize each class through different approaches. For example, one of CheNER’s configurations uses individual CRF recognizers for recognizing SYSTEMATIC, TRIVIAL, FAMILY, ABBREVIATION and FORMULA classes, and the configuration combines these recognizers with a regular expression-based IDENTIFIER recognition. This configuration achieves its best performance in the chemical document indexing (CDI) task (document-level CNR evaluation metric). In the chemical entity mention recognition (CEM) task (instance-level evaluation metric), its best configuration uses the IOB tag scheme and only one chemical class. Dai *et al*. (2) compared different tag schemes including IOB, BIOE, SOBIE and B1B2IOE, and showed that the SOBIE tag scheme seems to perform better in CNR. In general, both IOB and SOBIE tag sets are often used in state-of-the-art systems, and the single chemical label is most often used in the CNR task.

In the features set, the features used in the CNR task are similar with those used in other biomedical NER tasks. Normally, linguistic, orthographic, morphological and lexical features are used. However, additional features may be included to capture chemical names. For instance, CheNER has regular expression features to capture SYSTMATIC-class NEs, and Batista-Navarro *et al*. use five dictionaries including the Chemical Entities of Biological Interest (ChEBI) database (17), DrugBank (18), Comparative Toxicogenomics Database (CTD) (19), PubChem Compound (8) and the Joint Chemical Dictionary (Jochem). Their dictionary feature is encoded in the begin-inside-outside (BIO) format.

## Methods

### System description

**Figure 1:**
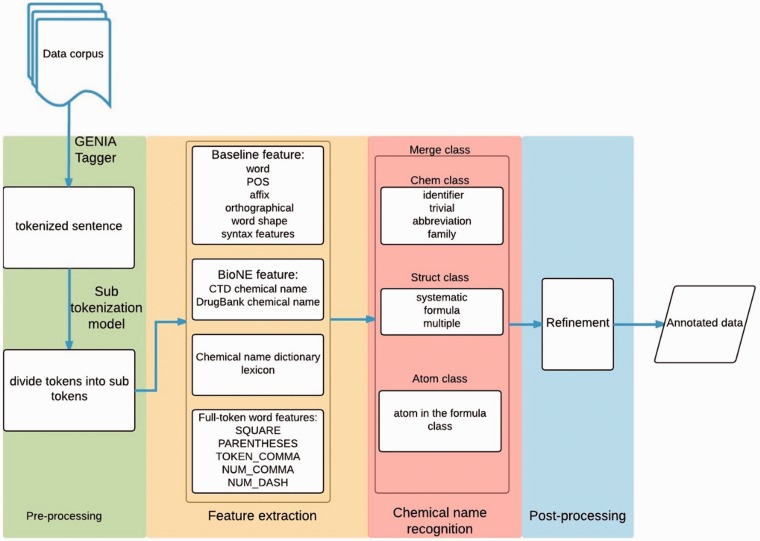
The workflow of our system.

Our approach involves four stages: pre-processing, feature extraction, chemical mention recognition, and post-processing. Figure 1 displays the workflow of the whole system. First, in the pre-processing stage, a rule-based method is employed to detect sentence boundaries in the given document. We use GENIATagger (20) to implement the rule-based method. After boundary detection, another rule-based approach is used to split the sentences into tokens. Second, we extract features including word, affix, orthographical, word-shape information, conjunction, biological named entity (Bio-NE) and full-token word features. Third, the chemical mention recognition stage exploits the extracted features to classify chemical mentions using the CRF model. Finally, our post-processing module scans the whole document to check the consistency of the results. The following sub-sections describe each stage in detail.

### Pre-processing

**Figure 2:**
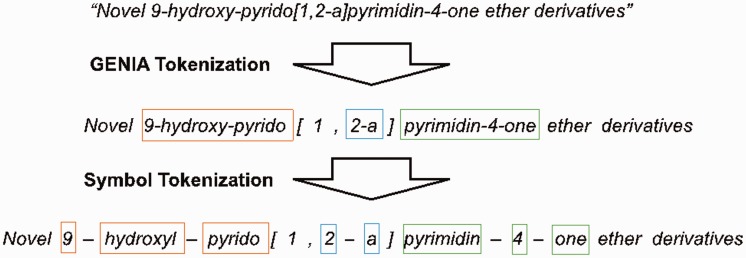
An example of tokenization.

We employ the GENIATagger to tokenize sentences into full tokens. Then, we run a sub-tokenization module used in our previous work (2) to further divide the tokens into sub-tokens. The main problem of basic GENIA tokenization for this application is that it does not break chemical expressions into small enough segments for our NE recognizer. For example, *pyrimdin* and ‘Novel 9-hydroxy-pyrido[1,2-a]pyrimidin-4-one ether derivatives’ are segmented as individual tokens by GENIA. However, if only *pyrimdin* appears in the training set, and ‘9-hydroxy-pyrido[1,2-a]pyrimidin-4-one ether derivatives*’* appears in the test set, our chemical NE recognizer is likely to label it incorrectly because ‘9-hydroxy-pyrido[1,2-a]pyrimidin-4-one ether derivative*’* is not learned during training. To solve this problem, we apply an extra sub-tokenizer that uses punctuation marks as delimiters (e.g. hyphens) to further segment expressions into sub-tokens. For instance, ‘9-hydroxy-pyrido[1,2-a]pyrimidin-4-one ether derivative’ would be segmented as *9*, *hydroxy*, *pyrido*, *1*, *2*, *a*, *pyrimdin*, *4*, “one”, *ether* and *derivatives*. As a result, there are fewer unseen tokens after extra tokenization. A tokenization example is demonstrated in Figure 2, in which the color borders indicate the stage in Figure 1 that the tokens were derived from.

### Feature extraction

**Figure 3:**
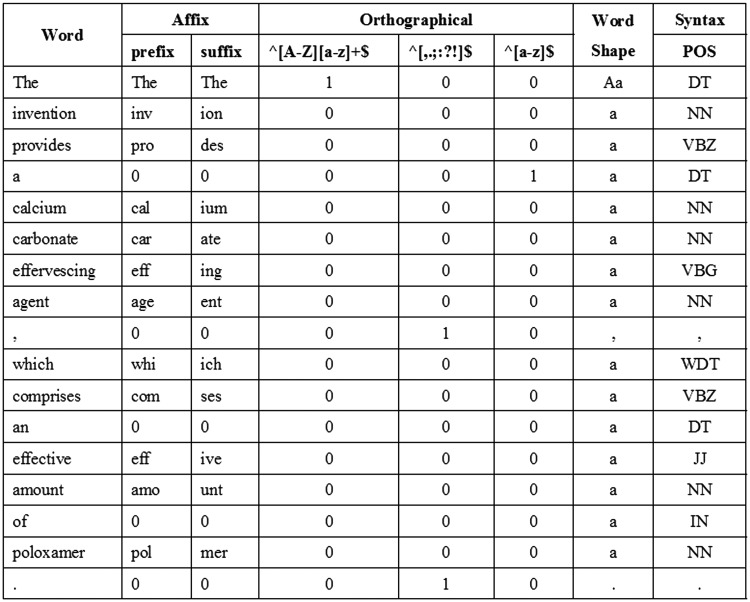
An example of the baseline features.

We utilize features from our previous work (2) including word, POS, affix, orthographical, word shape and syntax features as the baseline features. For word features, we normalize every single digit letter to ‘1’. In addition, since our extra tokenization step may sometimes impair feature information by over segmenting some expressions, we use the full GENIA tokens to extract surface features. Figure 3 shows an example of the baseline features corresponding to a sentence.

### NE features

#### Bio-NE

We use GENIATagger to recognize BNEs including DNA, RNA, protein, cell_line and cell_type. The presence of these BNEs is used as a feature. The labels predicted by GENIATagger are used as feature values.

#### Chemical name dictionary lexicon

We employ external dictionaries and a maximum matching algorithm to recognize chemical mentions from the Comparative Toxicogenomics Database (CTD). The CTD (19) chemical dictionary and a DrugBank (18) drug dictionary are used to recognize CTD chemical mentions and drug mentions. There are a total of three features: CTD chemical name feature, drug name feature, and chemical/drug name dictionary. The dictionary type is used as a feature value.

#### GENIA full-token word features

The full-token word features include the five orthographical features listed in Table 2 and one boundary feature.

**Table 2:** Chemical orthographical features

Feature name	Regular expression
SQUARE	\[.*?\]
PARENTHESES	\(.*?\)
TOKEN_COMMA	\S+,\S+
NUM_COMMA	\d,\d
NUM_DASH	\d-\d

**Figure 4:**
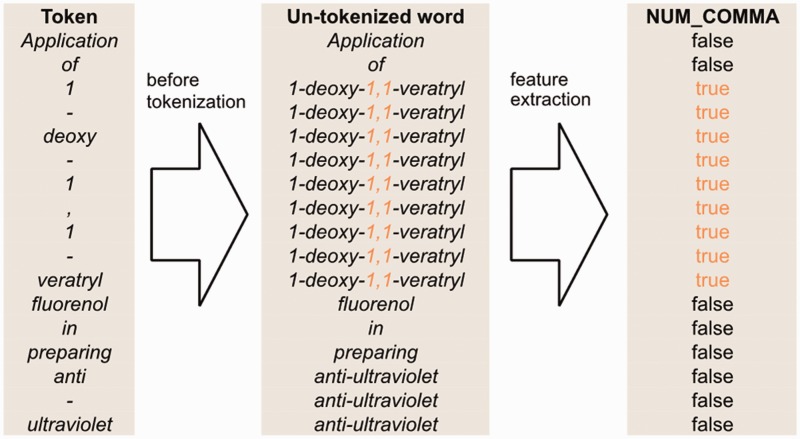
An example of un-tokenized word orthographical features for the sentence ‘Application of 1-deoxy-1,1-veratryl fluorenol in preparing anti-ultraviolet’.

Figure 4 illustrates how we generate the full-token word features. First, we use spaces as the delimiter to segment a sentence into a sequence of words. Then, we map each token to a word segmented in the previous step. However, if the token is a punctuation mark or the prefix or suffix of the word containing it, the token is mapped to itself. Afterwards, we apply our full-token orthographical patterns to match the original word. If matched, then the feature value will be true. Otherwise, it is false.

**Figure 5:**
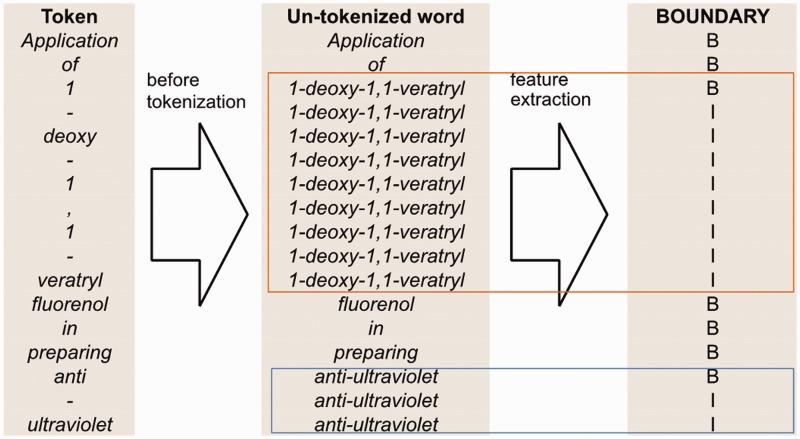
An example of boundary feature for the sentence ‘Application of 1-deoxy-1,1-veratryl fluorenol in preparing anti-ultraviolet’.

The boundary feature illustrated in Figure 5 is based on full-token boundaries. We use B to represent the beginning of a full token and I to represent an internal component of a token.

### Chemical name recognition

**Figure 6:**
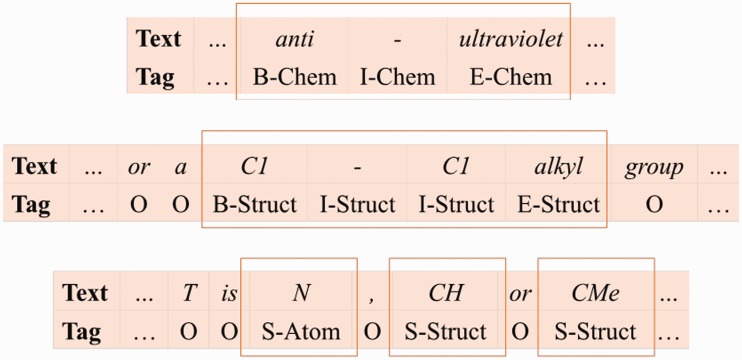
Examples of our label set.

Based on their naming conventions, we merge the seven chemical name type classes into three: (1) Chem class represents the identifier, family, trivial, and abbreviation classes; (2) Struct class represents the systematic, formula, and multiple classes; (3) Atom represents the atoms in the formula class. We use the linear chain CRF model (21) and SOBIE-label scheme (2) to combine the classes with S (singleton), B (beginning), I (inside), E (ended) and O (outside) to represent token labels. Examples of our label set are shown in Figure 6.

### Refinement

In the refinement step, we collect all chemical mentions excluding atoms recognized in a given document by our CRF-based recognizer, and use a maximum matching algorithm to check for missed instances. If a chemical mention overlaps with a previously recognized mention, the latter will be identified as the chemical name.

## Results

Our experiments were conducted on the Biocreative V CHEMDNER corpus. F1-measure is used as our evaluation strategy, which requires the start and end indices corresponding to the chemical entities. We reported the performance of three experiments. The first examined the effect of chemical concept composition. Then we observed the influence of adding different features such as BioNE, un-tokenized orthographical and boundary features. Finally, the performances of our submissions are reported.

### Dataset

The CHEMDNER patent corpus is used to evaluate our approach. It contains patents from 2005 to 2014 that have been assigned either theA61P^1^ or A61K31 ^2^IPC (International Patent Classification) codes, meaning the patents are relevant to medical chemistry and mention synthetic organics.

The CHEMDNER patent corpus is divided into the training, development, and test sets. Each set contains 7000 patent abstracts. An instance-based evaluation is applied for the Chemical Entity Mention in Patents (CEMP) subtask, indicating that both the mention span and class should be correct. Evaluation metrics are given as micro-averaged recall, precision and F-score.

### Effect of the chemical concept composition on the development set

We examined the effect of adding the Atom and Struct class. Results displayed in Table 3 shows that the addition of the Atom class improved the F-score by 0.5%, and adding the Struct class further improved it by 0.1%.

**Table 3:** CEMP run performances on the BioCreative V CHEMDNER-patents track development set

Configuration	CEMP		
	Recall	Precision	*F*-score
Chem	87.10	84.10	84.08
Chem+Atom	88.19	83.96	84.58
Chem+Struct+Atom	88.04	84.25	84.68

In scientific literatures, formulae such as ‘–CH = CH_2_’, ‘Ag+ ions’ and ‘Cu(C_10_H_12_NO_2_)_2_’ were not always annotated as ground truths. However, more than ∼35% of the formulae were annotated as the ground truths in the patents. Therefore, to enhance formulae recognition, we used the Atom class for recognizing chemical entities like ‘H’, ‘Cu’ and ‘Ag’, and added some chemical orthographic features into NERChem. We improve the F-score by 6% by recognizing these formulae.

### Effect of the features on the development set

We first explored the effect of Bio-NE feature on the performance by adding it to the baseline features. Subsequently, we observed the effect of un-tokenized orthographical features and boundary feature. Finally, we examine the performance of the system when all features and the refinement method are included. The results are listed in Table 4. The Bio-NE features improved the F-score by 0.44%, and adding the un-tokenized orthographical features and the boundary feature further improved the performance by 0.27 and 0.15%, respectively.

**Table 4:** The effect of the features on the CEMP run on the BioCreative V CHEMDNER-patents track development set

Run	Configuration	CEMP		
		Recall	Precision	*F*-score
1	Baseline features	87.10	84.10	84.08
2	1 + BioNE	87.22	85.28	84.52
3	2 + Untokenized Ortho.	88.13	84.36	84.79
4	3 + Untokenized Bound.	88.92	83.98	84.94

### Performance on the test set

We participated in both the CEMP and CPD subtask of the BioCreative V CHEMDNER-patents track. Table 5 displays the configurations and performances of our runs for CEMP. We achieved an F-score of 87.17% on CEMP, which is ranked 4th overall, and the best sensitivity of 98.576% on CPD that is in the 2nd place overall. Run 4 in Table 5 is the best configuration of our system which included the refinement, and we compared it with the performance of other participants and the average performance of 21 teams in Table 6 and Table 7. We found that some composite names such as *(C1–C4) alkoxy methyl*, *C4–C8 alcohol* and *C6–C9 aroyl* were misidentified in our system. However, SimConcept (22) focused on decomposing these mentions and was able to successfully recognize them. These cases were overlooked in our previous work, and we will seek to improve our system based on the approach of SimConcept.

**Table 5:** Performances of our runs for CEMP on the BioCreative V CHEMDNER-patents track test set

Run	Configuration	Recall	Precision	*F*-score
1	Chem + Atom label + BioNE	87.019	85.135	86.067
2	1 + refinement	87.181	85.385	86.274
3	2 + Untokenized Word	87.852	86.065	86.95
4	3 + Untokenized Bound.(best)	88.86	85.534	87.165

**Table 6:** Comparison of CEMP performances of other participants with our system on the CHEMDNER-patents test set

Rank	Team-Id	Recall	Precision	*F*-score
1	274	91.293	87.517	89.366
2	288	90.777	87.177	88.941
3	362	89.375	86.885	87.778
4	356 (our run 4 in Table 5)	88.86	85.534	87.165
–	average	81.159	78.061	79.472

**Table 7:** Comparison of CPD performances of other participants with our system on the CHEMDNER-patents test set

Team-Id	Sens.	Spec.	Accur.	MCC	P_full_R	AUC_PR
288	98.598	87.209	94.75	88.237	66.571	93.468
356 (our run 4 in Table 5)	98.576	83.425	93.457	85.362	66.214	92.119
276	98.166	85.877	94.014	86.556	66.219	93.042
313	97.875	59.429	93.464	65.99	66.314	89.211
average	87.516	86.904	87.310	74.899	66.228	92.838

## Conclusion

This study proposes a method that aims to automatically recognize chemical compounds in chemical patents. The method uses the same tokenization features and tag sets in our previous work (2) on the BioCreative IV CHEMDNER corpus. Additionally, we use the chemical class composition to combine classes with similar naming convention, and the result shows that it outperforms our previous approach, which merged all chemical classes into one. Furthermore, we added the Bio-NE and un-tokenized word features, and demonstrated their effect on system performance. Our approach is also evaluated on the BioCreative V CHEMDNER-patent task. Applying the refinement method as the post-processing step, we achieved an F-score of 87.17% on CEMP, which ranked 4th overall, and obtained the best sensitivity of 98.576% on CPD that is in the 2nd place overall.

*Conflict of interest*. None declared.

## Funding

This work was supported by the National Science Council of Taiwan (103-2221-E-008-044-MY3). Funding for open access charge: National Science Council of Taiwan Grant No. 103-2221-E-008-044-MY3.

## Footnotes

1 Specific therapeutic activity of chemical compounds or medicinal preparations.

2 Medicinal preparations containing organic active ingredients.
